# Revision total knee arthroplasty (TKA): mid-term outcomes and bone loss/quality evaluation and treatment

**DOI:** 10.1186/s13018-019-1328-1

**Published:** 2019-08-28

**Authors:** Federica Rosso, Umberto Cottino, Federico Dettoni, Matteo Bruzzone, Davide Edoardo Bonasia, Roberto Rossi

**Affiliations:** 1 Department of Orthopedics and Traumatology, AO Ordine Mauriziano, Largo Turati 62, 10128 Turin, Italy; 2 Department of Surgery, University of Turin, Via Po 8, 10100 Turin, Italy

**Keywords:** Knee, Revision arthroplasty, Outcomes, Bone defects, Classification

## Abstract

**Background:**

Revision total knee arthroplasty (rTKA) is a demanding procedure, with a high complication and failure rate and a high rate of bone losses and poor bone quality. Different classifications for bone losses have been proposed, but they do not consider bone quality, which may affect implant fixation. The aim of this study is to describe the outcomes of a consecutive series of rTKA. Furthermore, a modified bone loss classification will be proposed based also on bone quality. Finally, the association between radiolucent line (RLL) development and different risk factors will be evaluated.

**Methods:**

All the patients who underwent rTKA between 2008 and 2016 in the same institution were included. rTKAs were performed by the same surgeon according to the three-step technique. Bone losses were classified according to the proposed classification, including bone quality evaluation. The Knee Scoring System (KSS), the Hospital for Special Surgery Knee Score (HSS), and the SF-12 were used for the clinical evaluation. Radiological evaluation was performed according to the Knee Society Roentgenographic Evaluation System. Different possible risk factors (i.e., gender, age, amount of bone losses) associated to RLL development were identified, and this association was evaluated using logistic regression.

**Results:**

Fifty-one patients (53 knees) were included (60.8% female, average age 71.5 years). The average follow-up was 56.6 months (range 24–182). The most frequent cause of failure was aseptic loosening (41.5%). 18.9% of the cases demonstrated poor bone quality. Bone losses were treated according to the proposed algorithm. In all the cases, there was a significant improvement in all the scores (*P* < 0.05). The average post-operative range of motion was 110.5° (SD 10.7). At the radiological evaluation, all the implants resulted well aligned, with 15.1% of non-progressive RLL. There were 2 failures, with a cumulative survivorship of 92.1% at the last follow-up (SD 5.3%). At the logistic regression, none of the evaluated variables resulted associated to RLL development.

**Conclusion:**

rTKA is a demanding procedure, and adequate treatment of bone losses is mandatory to achieve good results. However, also bone quality should be taken into consideration when approaching bone losses, and the proposed classification may need surgeons after an adequate validation.

**Level of evidence:**

Level IV

## Background

The number of total knee arthroplasty (TKA) procedures is growing worldwide, with an expected future increase of 143% by 2050 [1]. Good outcomes are described in the literature, with a survivorship of primary TKA ranging between 90% and 95% at 15-year follow-up [2]. Considering these data, and the increased number of patients at higher risk of TKA failure (i.e., younger patients) [3], the concomitant increase in the incidence of revision TKA (rTKA) procedures is not surprising. Some authors estimated that the number of rTKA will increase by 600% by 2030 [4]. Unfortunately, the survivorship of rTKA is inferior compared to primary TKA, ranging from 71 to 86% at 10-year follow-up [2, 5]. Adequate implant fixation accounting for bone loss amount and bone quality is paramount to improve implant survivorship. Morgan Jones et al. described “zonal” fixation in rTKA, considering three zones: epiphysis, metaphysis, and diaphysis. The authors concluded that good fixation should be achieved in at least 2 zones in rTKA [6]. Bone loss in rTKA has been historically classified according to the Anderson Orthopaedics Research Institute (AORI) classification, which considers the location of bone loss and defect size [7]. Different authors described the available options to treat bone losses in rTKA which include cement, impaction bone grafting, traditional metal augments, structural allograft, metal cones, or sleeves [8, 9]. However, some authors reported high mid-term failure rates using cement, morselized, or structural bone allograft, probably due to poor bone quality in the metaphysis [10, 11]. In these cases, Trabecular Metal^TM^ tantalum cones (Zimmer, Warsaw, IN, USA) can be used to reconstruct metaphyseal bone defects (2A or greater) and to improve implant fixation [12–14]. Furthermore, in most rTKA (particularly after septic loosening), the bone quality is very poor and sclerotic, and it may be useful to add metaphyseal fixation with these cones to obtain more solid implant fixation.

The aim of this study was to evaluate the outcomes of a consecutive series of rTKA in which implant fixation has been obtained in at least two zones [6]. Furthermore, rTKA and bone loss evaluation was performed according to a modified bone loss classification system, based on the one described by Engh [7], but taking into consideration also the bone quality evaluation together. Lastly, possible risk factors for the development of radiolucent lines, including the presence of additional fixation in the metaphyseal zone, will be evaluated.

## Material and methods

### Patients’ demographics and evaluation

This is a single-center prospective study of a consecutive series of rTKA performed at our institution between January 2008 and 2016 by the senior author (RR). Inclusion criteria were rTKA performed for any reason by the same surgeon (including re-revision), complete revision (on both the femoral and tibial side), and a minimum follow-up of 24 months. Exclusion criteria were revision from unicompartmental knee arthroplasty to total knee arthroplasty or use of any so-called mega-prosthesis. After registry evaluation, 53 patients matched the inclusion criteria and were included in the study.

All patients were evaluated pre-operatively, including assessment for periprosthetic joint infection (PJI). All patients underwent serum ESR and CRP evaluation, and if significantly elevated according to the criteria proposed by Parvizi et al., joint aspiration was performed to evaluate white cells’ count and polymorphonucleate percentage [15, 16]. Radiographic evaluation including long leg, standing anteroposterior, lateral, and Merchant views were performed to evaluate for aseptic loosening, patello-femoral disorders (i.e., patella baja), extensor mechanism rupture, implant failure, malalignment, or periprosthetic fracture [17, 18]. CT scan was performed in selected patients when component malrotation was suspected or if more accurate evaluation of bone loss was required [18]. Clinical evaluation was performed focusing on tibiofemoral stability, patellofemoral tracking, and range-of-motion (ROM). Stiffness was defined as a ROM below to 70° while ankylosis was defined as ROM below to 30° [19]. The cause of failure was classified, based on the pre-operative evaluation, according to the list proposed by Vince [20]: (1) aseptic loosening, (2) instability, (3) patellar complications and malrotation, (4) structural failure of the implant, (5) PJI, (6) extensor mechanism rupture, (7) stiffness, (8) periprosthetic fracture, and (9) no diagnosis, the so-called mystery knee.

### Surgery-related data

A semi-constrained or rotating-hinged implant was used depending on the amount of bone loss and ligamentous deficiency. The indications for a rotating hinged implant were (1) disruption of both collateral ligaments or one collateral ligament in combination with posterior capsule disruption, (2) not correctable flexion/extension mismatch, (3) dislocation, (4) extensor mechanism insufficiency, (5) neuromuscular condition, and (6) revision of a previous rotating hinged implant [21]. In case of PJI, a two-stage revision was performed using mobile or static cement spacers, depending on the presence of severe ligamentous instability, insufficient extensor mechanism, massive bone loss, or compromised soft tissue [15, 22]. The second stage was performed once the clinical and laboratories tests were negative for infection.

All the patients underwent a rTKA according to the “three-step technique” proposed by Kelly Vince [23]. In all the cases, the tourniquet was used only during cementation. All the implants were cemented using antibiotic-loaded cement. Tranexamic acid was used to reduce blood loss in all the cases. Femoral or tibial stems were used in all the cases to achieve a good diaphyseal fixation [24]. Offset stems were used in case of (1) anatomical mismatch between the center of the metaphysis and the center of the diaphysis, (2) need for malalignment correction, and (3) need to improve gap balancing (in order to reduce flexion gap or to avoid femoral notching) [24].

**Fig. 1:**
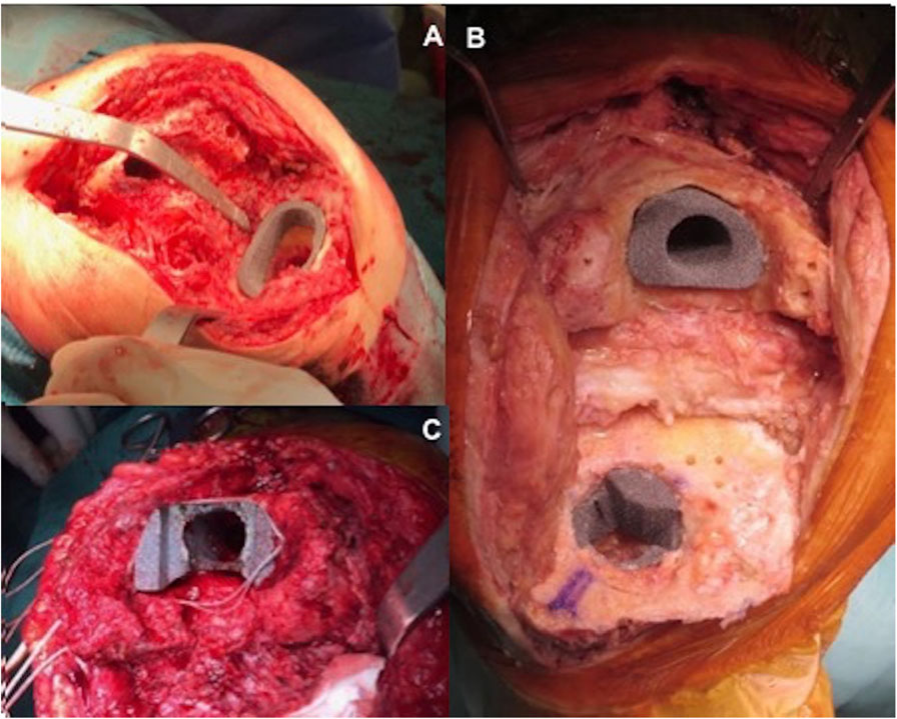
Intra-operative pictures demonstrating differences in bone quality: **a** good bone quality, **b** sclerotic bone quality, and **c** osteoporotic bone quality

Bone loss was initially classified according to the AORI classification [7]. However, this classification was slightly modified in this study introducing bone quality evaluation into the methodology. Specifically, bone quality in the epiphysis and metaphysis was classified as good (G) if the bone structure was strong enough with good cancellous bone and good bleeding after bone preparation (Fig. 1a). This type was seen in most of the cases of revision for instability, patellofemoral disorders, or malalignment. The second type of bone quality was defined as “sclerotic (S)” if there was not good bleeding after bone preparation in association with the absence of the trabecular structure of the cancellous bone that had the typical “marble aspect” (Fig. 1b). This type is typical after the use of cement spacer for septic loosening, in cases of multiple revisions or in prolonged aseptic loosening. Lastly, bone quality can be classified as “osteoporotic (O)” in the presence of good bleeding after bone preparation but with an increase in the porous size of the trabecular structure of the cancellous bone with poor bone quality (bone collapse after finger pression) (Fig. 1c). This was mostly present in older patients or patients affected by inflammatory disease or chronic renal disease. Table 1 shows the modified AORI classification.

**Table 1:** AORI classification modified according to authors proposal based on bone quality

Femoral (F) or tibial (T) grading	Description
1	(INTACT metaphyseal bone): Minor bone defects that do not compromise the stability of the component. • 1G: Good bone quality • 1S: Sclerotic bone quality • 1O: Osteoporotic bone quality
2	(DAMAGED metaphyseal bone): Cancellous bone loss requiring cement fill, augments, or bone graft to restore a reasonable joint line level. • 2A➔One femoral condyle or tibial plateau • 2AG: Good bone quality • 2AS: Sclerotic bone quality • 2AO: Osteoporotic bone quality
	• 2B➔Both femoral condyle or tibial plateau • 2BG: Good bone quality • 2BS: Sclerotic bone quality • 2BO: Osteoporotic bone quality
3	(DEFICIENT metaphyseal segment): Bone loss with a defect of a major portion of condyle or tibial plateau. May involve ligament attachments. • 3G: Good bone quality • 3S: Sclerotic bone quality • 3O: Osteoporotic bone quality

Bone loss was treated according to previous studies using cement, impaction bone grafting, traditional metal augments, or tantalum cones [8, 9]. However, in cases of sclerotic or osteoporotic bone, acceptable implant fixation in the metaphysis could not be obtained with these methods. In these cases, some form of metaphyseal fixation (i.e., tantalum cone) was useful to enhance implant fixation and to avoid early aseptic loosening, despite sacrificing some host metaphyseal bone. The thought process applied to treat the bone loss, according to these principles, is shown in Table 2.

**Table 2:** Decisional algorithm according to the modified AORI classification

AORI	Bone quality	Treatment option
F1-T1	Good (G)	• < 5 mm (< 50% of bone surface area)➔Cement and morselized bone • 5–10 mm➔Cement and screw or morselized bone
	Sclerotic (S) or osteoporotic (O)	• Be sure to obtain adequate zone 3 (diaphysis) fixation • If very sclerotic bone, consider small tantalum cone (disadvantage is to sacrifice bone stock
F2A-T2A	Good (G)	• 5–10 mm➔Cement and screw only if low demand patients • > 5 mm; > 40% of surface unsupported from host bone➔Metal augments or structural allograft or impaction bone grafting (young patients) • Need for adequate zone 3 fixation (stems)
	Sclerotic (S) or osteoporotic (O)	• Same option than before • Adequate zone 2 fixation (cone) strongly recommended to reduce risk for aseptic loosening
F2B-T2B or type 3 defect	Good (G)	• Impaction bone grafting (young patients), metal augments, structural allograft • Larger defect➔tantalum cone and titanium sleeve with short to medium-length stems • Severe type 3 defect➔mega prosthesis
	Sclerotic (S) or osteoporotic (O)	• Same option than before • Tantalum cone is often required to contemporarily treat bone loss and to enhance zone 2 fixation

### Clinical and radiological evaluation

The Knee Scoring System (KSS), the Hospital for Special Surgery Knee Score (HSS) [25], and the SF-12 [26] outcome measures were utilized. All the patients were clinically and radiographically evaluated in 2018.

All the patients underwent pre- and post-operative evaluation of limb alignment, component positioning, and presence of a radiolucent line (progressive or not) evaluation using the Knee Society Roentgenographic Evaluation System [27].

### Statistical analysis

Descriptive statistic was used for all demographic, subjective, and objective outcomes. Data was collected with an Excel® spreadsheet (Microsoft, Redmond, WA, USA) and presented with average and standard deviation (SD). *T* test and *χ*^2^ test were used to analyze differences in, respectively, continuous and categorical variables. The Kaplan-Meier method was used to evaluate cumulative survivorship. Possible risk factors for radiolucent line (RLL) development were identified (i.e., gender, older age (> 75 years), increased body mass index (BMI > 30 kg/m^2^), hinged implant, use of augments, augment height (> 5 mm), bone loss above 2B, and poor bone quality defined as severely osteoporotic or sclerotic). Each variable was firstly tested in a simple regression model to evaluate the association with the presence of RLL. All the variables with *P* < 0.1 were then re-tested in a multiple regression model to identify possible risk factors associated to RLL development.

### The Institutional Review Board (IRB) approval

The study is performed in accordance with the ethical standards in the 1964 Declaration of Helsinki and with the HIPAA regulation. The Institutional Review Board (IRB) of the author’s institution defined this study as exempt from IRB approval (prospective study on a well-established surgical procedure). Verbal informed consent to participate at the study was obtained for every included patient.

#### Results

**Fig. 2:**
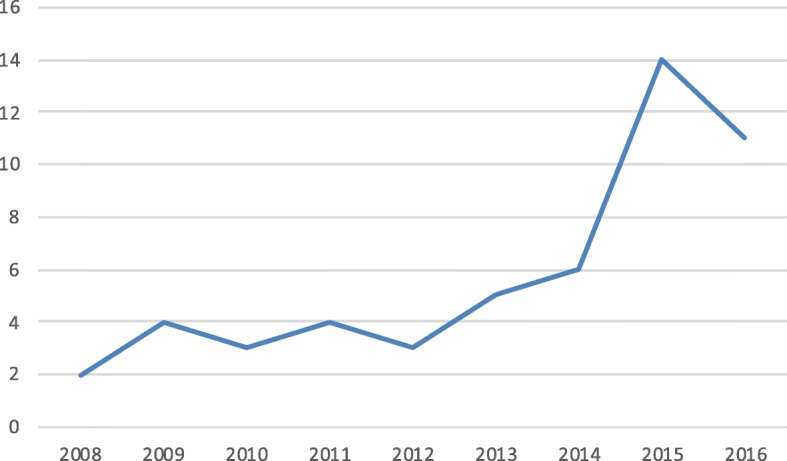
Graph including numbers of revision TKA performed each year with an increase in the last 2 years

Fifty rTKA in 51 patients were included in the study, with none lost to follow-up and an average follow-up of 56.6 months (SD 35.6 months, range 24–182 months). There was an increase in the number of rTKA from 2008–2016, as shown in Fig. 2. There were 31 females (60.8%) and 20 males (39.2%), with an average age of 71.5 years (SD 8.8 years) and 35.8% of patients > 75 years old. The average body mass index (BMI) was 28.7 kg/m^2^ (SD 5.6).

**Fig. 3:**
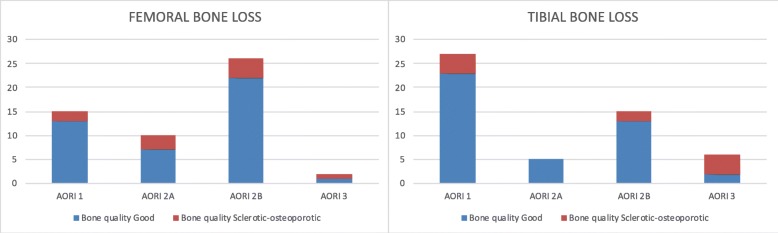
Graph demonstrating the distribution of bone loss according to the modified AORI classification

The most frequent cause of failure was aseptic loosening (41.5%), followed by septic loosening (30.2%), instability and the so-called mystery knees (9.4% each), stiffness (7.6%), and extensor mechanism insufficiency (1.9%). Particularly, this last patient had a loose implant associated to an extensor mechanism disruption, and a reconstruction of the extensor mechanism using mesh, as described by Brown and Hanssen, was performed together with the revision TKA [28]. In 69.8% of patients, a condylar constrained implant was used, while in the remaining cases, a rotating hinged implant was necessary. Bone loss was classified according to the modified AORI classification as shown in Fig. 3. Ten cases (18.9%) were considered having poor bone quality (nine cases of septic loosening and one case of multiple revisions, seven cases of sclerotic bone, and three cases of severe osteoporotic) on both the femoral and tibial side. In the remaining 43 patients, the bone quality was classified as were good (81.1%). Furthermore, on the femoral side in 52,8% of the cases, the bone loss was classified as 2B or more, compared to 39.6% on the tibial side. In 84.6% of the cases at least one augment was used to address the bone loss or to restore joint line height. One hundred ten traditional metal augments were used on the femoral side in 53 cases (distal medial, distal lateral, posterior medial, and lateral), with 19.1% of augments greater than 5 mm. In all the cases a femoral stem was used. The most commonly used was the 100-mm-long stem (73.6%), and all the stems were diaphyseal-engaging cementless stems. An offset stem was used in 16 cases (30.2%): anteriorizing in 7 cases, posteriorizing in 5 cases, medializing in 3 cases and lateralizing in 1 case. A femoral cone was used in 2 cases, and in both the cases, it was necessary because of severe bone loss (greater than 2B) and poor bone quality (sclerotic or severe osteoporotic bone). On the tibial side, 40 augments were used (only medial or lateral) with 32.5% of augments greater than 5 mm. On the tibial side, in 2 cases, the tibial stem was not used because the implant size and model did not allow using a stem. The most commonly used stem was the 100-mm-long one (32.1%); in 5 cases, a short-cemented stem was used (9.4%) and offset was used in 5 cases (9.4%) to correct medio-lateral position of the tibial tray. On the tibial side, a tantalum cone was implanted in 6 cases, with 2 cases of bone loss but good quality and 4 cases of bone loss and sclerotic bone quality. In the remaining cases with poor bone quality (sclerotic or severe osteoporotic), a tantalum cone was not implanted because it was not yet available at our institution.

ROM significantly improved from 101.7° (SD 20.2) preoperatively to 110.5° (SD 10.8) at final follow-up (*P* = 0.005). The KSS significantly improved from 69.6 points (SD 15.2) preoperatively to 79.6 points (SD 24.9) at final follow-up (*P* < 0.001). Furthermore, the average postoperative KSS expectation (in respect to rTKA outcome) was 9.04 (SD 1.16) and the KSS satisfaction was 30.3 (SD 5.2), demonstrating good patient satisfaction. Lastly, the HSS score also significantly improved from 67.4 points (SD 10.7) preoperatively to 82.5 points (SD 8.4) postoperatively. The SF-12 demonstrated good scores in both physical (PCS) and mental (MCS) section, 44.2 points (SD 6.2) and 47.8 points (SD 4.8), respectively).

Postoperative complications occurred in 5.6% of the patients, with 3.8% quadriceps tendon lesion related to a trauma (following to a fall) and 1.9% superficial infection. There were no cases of recurrent infection in patients who underwent revision for septic loosening. There were no cases of deep vein thrombosis or pulmonary embolism.

**Fig. 4:**
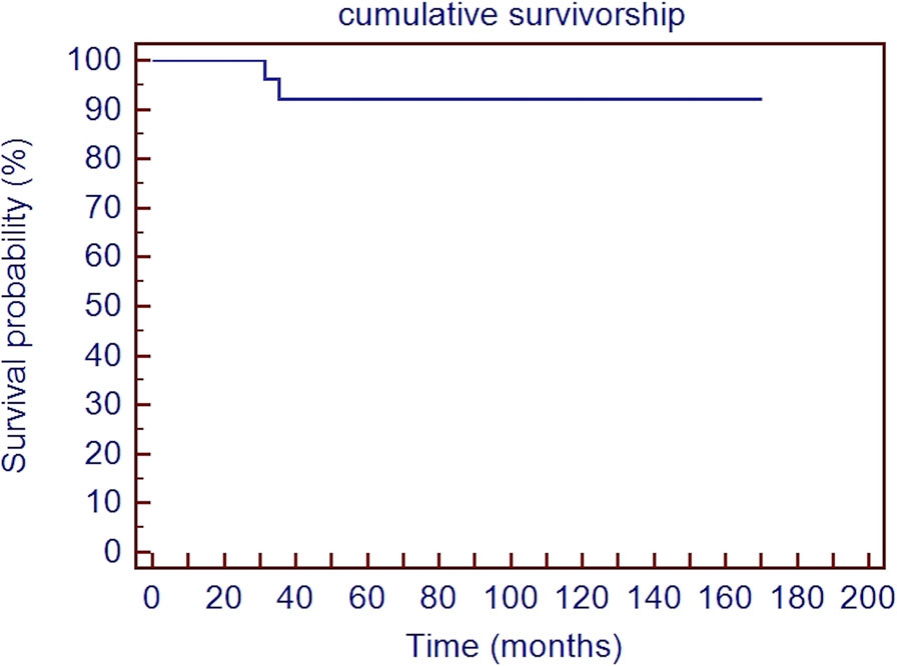
Cumulative survivorship calculated using the Kaplan-Meier method

Failure was considered as re-revision TKA. With this end-point, there were two failures. One patient sustained femoral component loosening associated with failure of the hinge mechanism 3 years after the revision and underwent femoral component revision using a tantalum cone. The second patient underwent femoral component revision due to a periprosthetic fracture 3 years after the first revision. The cumulative survivorship was 92 1% at the last follow-up (SD 5.3%), as shown in Fig. 4.

All the implants were well aligned radiographically with average *α* angle 94.2° (SD 1.3), average *β* angle 90.4° (SD 1.2°), average *γ* angle 3.8° (SD 0.4), and average *δ* angle 86.5° (SD 1.8). The average joint line height, measured from epicondyle to joint line, was 26.8 mm (SD 2.4). Eight cases (15.1%) demonstrated radiolucent lines greater than 2 mm, mostly located on the tibial side on zone 5–6 and 7 on the AP view (5 cases 62.5%). The remaining RLLs were located at the femur, particularly 2 cases in zone 1 and 1 case in zones 1 and 2 on the lateral view. However, in no cases where a tantalum cone was used were radiolucent lines detected at final follow-up. Using logistic regression, none of the variables we considered were associated with the presence of radiolucent lines greater than 2 mm (Table 3).

**Table 3:** Summary of the simple and logistic regressions performed to evaluate the possible risk factors associated to radiolucent lines development (N/A=not applicable)

	RLL Tibia				RLL femore			
	Simple test	OR	IC95%	*P* value	Simple test	OR	IC95%	*P* value
Gender (female)	0.355	N/A			0.154	N/A		
Age (> 75 years)	0.031	8.8000	0.9048–85.5849	0.0610	0.189	N/A		
BMI (> 30 Kg/m^2^)	0.582	N/A			0.206	N/A		
Hinged (yes)	0.672	N/A			0.134	N/A		
Augments	0.441	N/A			0.400	N/A		
Augments height (> 5 mm)	0.645	N/A			0.905	N/A		
Tantalum cone	0.529	N/A			0.730	N/A		
Bone loss > 2B	0.337	N/A			0.496	N/A		
Poor bone quality (yes)	0.624	N/A			0.249	N/A		

## Discussion

This is a prospective study including 53 rTKA performed in 51 patients by the same surgeon at an average follow-up of 56.6 months.

The first finding of the study was that the most frequent cause of TKA failure in this series was aseptic loosening (41.5%), followed by septic loosening (30.2%). This is similar to other published reports [29, 30]. The second finding of this study was that rTKA is a complex surgery, with a relatively high complication rate (6%), similarly to the results of other case series [31].

However, if the revision is performed following a step-wise approach, such as the three-step technique [23] and the level of constraint is accurately chosen based on bone loss and ligamentous insufficiency [32] good mid-term clinical and radiological outcomes may be obtained [21, 33]. A mid-term cumulative survivorship of 92.1% was reported in this series at the last follow-up. It can be considered a good mid-term survivorship, also compared to other studies in literature [33, 34].

One of the main problems challenging the surgeon in rTKA is the evaluation and treatment of bone loss. The most used system to evaluate bone loss is the AORI classification by Engh et al. [7], which considers the amount and location of bone loss. However, this classification does not account for bone quality. Different authors described the risk of developing radiolucent lines and bone resorption with full cementation in total knee arthroplasty [35]. Other authors described an increased risk of radiolucent lines and aseptic loosening with metal augments in revision TKA, especially in the presence of sclerotic bone [36–38]. For these reasons, a new classification for bone loss, which considers also bone quality, as the one proposed by the authors (Table 1), should be validated and introduced. Bone loss may be treated with different options, depending on the severity of the defect and the quality of bone losses, including cement, impaction bone graft, traditional metal augments, structural allograft, tantalum cones, or sleeves [8, 9]. Particularly, tantalum cones have been relatively recently introduced to treat major bone loss, with good outcomes [13, 39, 40]. Different authors described the biomechanical properties of tantalum, including high biocompatibility, high density, and possibility of porous structure with increased osteoconductive properties [41]. For all these reasons and because of their osteoconductive and positive biological properties, tantalum cones may be also useful to achieve a good metaphyseal fixation in presence of poor bone quality one, allowing for a stable “zonal” fixation as previously described by Morgan [6, 42]. Furthermore, different authors confirmed that radiolucent lines development may be correlated to instability, micromotion, inadequate load distribution, and different of elasticity between bone and metal and that they may decrease using some material more similar to bone properties, such as tantalum [43].

All these aspects, including the amount of bone loss and bone quality, should be considered during rTKA. For this reason, when approaching to bone loss, the algorithm of treatment should consider location and amount of bone losses, bone quality, and need for solid “zonal” fixation, as the one proposed in this manuscript (Table 2). In this algorithm, tantalum cones may be also used to enhance implant fixation in the metaphyseal zone in the presence of sclerotic bone, to reduce the risk of aseptic loosening due to insufficient epiphyseal fixation, particularly at the tibial baseplate. The biological and mechanical properties of tantalum cones are well known [14]. Considering the osteoconductive properties of tantalum, it is reasonable to think that it may allow for a stronger fixation in the metaphyseal zone, reducing the forces on the epiphyseal zone and, consequently, the risk for aseptic loosening [12, 39, 44]. However, surgeons have to be careful because the internal diameter of the cone may limit the diameter of the stem, or the use of an offset, possibly resulting in suboptimal Canal Fill Ratio (CFR). However, some studies demonstrated that the cone is not an obstacle to obtain a good stem alignment and CFR [45].

In this case series, only one failure was due to implant loosening (only femoral component) associated with hinge breakage was reported. In this case, no metaphyseal fixation was used during the first revision. Despite the small numbers of cones, mostly due to the recent introduction of tantalum cones, overall good results in terms of implant fixation were obtained in patients with sclerotic bone. However, logistic regression analysis found no association between the examined variables and the development of radiolucent lines, including poor bone quality. This is probably due to the small number of patients included in the case series, so further studies are necessary to confirm the role on metaphyseal fixation systems, such as tantalum cones, in enhancing implant fixation in the presence of sclerotic bone quality.

This study has several limitations. Firstly, it is a small series (53 cases) with a medium-term follow-up (56 months, minimum follow-up of 24 months). However, the case series cannot be enlarged so far in order to guarantee a minimum acceptable follow-up. Furthermore, the proposed classification has not been validated with testing for inter-and intra-observer reliability. Further studies are necessary to validate this classification, including inter and intra-observer reliability compared to other available classification, so that it can be applicable for every surgeon. The authors were not able to find any variable associated with radiolucent line development, including poor bone quality or number of augments or any variable related to insufficient epiphyseal fixation and need for metaphyseal fixation. This may be due to the small number of patients with poor bone quality (sclerotic or severe osteoporotic) and the small number of tantalum cones implanted. Another limitation is the lack of a matched control group, so a comparison between patients with improved metaphyseal fixation or cannot be compared. Lastly, this case series included only one type of tantalum cone, but there are different solutions available, such as sleeves, which may be useful to treat bone loss and to improve implant fixation in the presence of poor bone quality and which have not been considered in this case series. Furthermore, in the presence of massive bone losses, also customized implant may be considered, and they are not included in this case series.

However, also considering these limitations, this new proposed classification may be a valuable instrument for the surgeon to evaluate not only bone loss but also bone quality and to choose the right method of fixation required to obtain adequate bone loss treatment and implant fixation.

## Conclusion

In conclusion, revision TKA is a demanding procedure for both the surgeon and the patient, but if a step-wise approach is used during surgery, bone loss is correctly evaluated and treated, and good implant fixation is obtained, good clinical and radiological outcomes may be achieved at mid-term follow-up. Considering the risk for aseptic loosening due to poor “zonal” fixation, bone losses should be classified also according to bone quality, and surgeons should evaluate the appropriate bone loss treatment also according to the possibility to obtain a strong implant fixation. For this reason, a new classification based also on bone quality is proposed in this manuscript. However, further studies are needed to validate this classification and to confirm better outcomes in terms of implant durability if metaphyseal fixation is added in the presence of poor, particularly sclerotic, bone quality.

## Data Availability

All data and materials regarding the study are available at the corresponding author.

## Abbreviations

AORI: Anderson Orthopaedics Research Institute
BMI: Body mass index
CFR: Canal Fill Ratio
G: Good bone quality
HSS: Hospital for Special Surgery Knee Score
IRB: Institutional Review Board
KSS: Knee Scoring System
MCS: Mental SF-12 section
O: Osteoporotic bone quality
PCS: Physical SF-12 section
PJI: Periprosthetic Joint Infection
RLL: Radiolucent lines
ROM: Range-of-motion
rTKA: Revision total knee arthroplasty
S: Sclerotic bone quality
SD: Standard deviation
TKA: Total knee arthroplasty
